# Sustained choroid plexus function in human elderly and Alzheimer’s disease patients

**DOI:** 10.1186/2045-8118-10-28

**Published:** 2013-09-24

**Authors:** Reynold Spector, Conrad E Johanson

**Affiliations:** 1 Department of Medicine, Robert Wood Johnson Medical School, 105 Stone Hill Road, Colts Neck, NJ 07722, USA; 2 Department of Neurosurgery, Alpert Medical School at Brown University, 593 Eddy Street, Providence, RI 02903, USA

**Keywords:** Ascorbic acid, Folate, Transthyretin, Albumin, Cerebrospinal fluid, CSF acid–base balance, CSF pH and bicarbonate, CSF K homeostasis, Methyltetrahydrofolate carrier, Reduced folate carrier, Na-K-Cl cotransporter, Sodium vitamin C transporter 2, Choroidal epithelium, Blood-CSF barrier, CSF vitamins, CSF formation rate

## Abstract

We and other investigators have postulated deterioration of essential choroid plexus (CP) functions in some elderly and especially Alzheimer’s disease patients based on apparent anatomical, histological and pathological changes in CP. We have termed this putative phenomenon CP failure. By focusing on four essential energy-requiring CP functions, specifically ascorbic acid (AA) and folate transport from blood into CSF, transthyretin synthesis and secretion into CSF, and electrolyte/acid–base balance in CSF, we were able to evaluate the hypothesis of CP failure by reviewing definitive human data. In both healthy elderly and Alzheimer’s disease patients, the CP functions normally to transport AA and folates actively from blood into CSF, synthesize and secrete transthyretin into CSF, and maintain CSF acid–base balance and ion concentrations. These human CSF compositional data provide no support for the notion of CP failure in elderly humans and Alzheimer’s disease patients.

## Introduction

The human choroid plexus (CP), a locus of the blood-CSF barrier (BCSFB), is an organ in the cerebral ventricles weighing approximately 2 grams [1]. The CP epithelium secretes most of the CSF and helps to maintain a stable extracellular environment for brain (homeostasis) [2]. The CP achieves these ends, in part, by clearing unwanted substances from the CSF [3,4] and by pumping required compounds including certain vitamins [e.g., ascorbic acid (AA) and methyltetrahydrofolate (folates)], hormones and peptides from blood into CSF [2,5]. From CSF, these substances penetrate into brain because there is a negligible barrier to penetration via diffusion at the CSF-brain interfaces [2,6]. CP epithelial cells also synthesize peptides and proteins, e.g., transthyretin, for secretion into CSF [7-9].

In order to perform these functions, the human CP has an estimated blood flow of 4 to 5 ml/min/g and normally produces CSF at a rate of ~ 0.4 ml/minute, although there is substantial diurnal variation (see below) [2]. The anatomical BCSFB to even small molecules like urea [10] is due to the tight junctions that join the single-layered CP epithelial cells [2] in the ventricles and the multi-layered arachnoid membrane cells in the subarachnoid space. Capillaries in CP are “leaky”, unlike the microvessels in most of the brain where capillary endothelial cells are joined by tight junctions: the blood–brain barrier (BBB) [2].

Soon after our work on the vitamin transport systems in animal CP almost forty years ago, one of us (RS) and colleagues proposed that the non-stroke related dementias were due to a central vitamin deficiency state secondary to CP failure–analogous to renal failure [11]. This hypothesis was based on the anatomical and structural changes in CP of the elderly, and especially, in demented patients [1]. How could such a severely damaged CP organ perform adequately in Alzheimer’s disease? Subsequently others refined and expanded this failing CP hypothesis [12,13], including considerations for aging animal models [14,15]. However, we now summarize human data demonstrating, in both the elderly and Alzheimer’s patients, that this hypothesis needs reconsideration. It is important to resolve such hypotheses so that more fruitful avenues of research can be pursued.

### Interpretation of the human data

In order to evaluate CP function in the elderly and Alzheimer’s disease patients, we focus on four essential functions of the CP–AA and folate transport from blood into CSF via the CP, transthyretin synthesis/secretion into CSF [14], and CSF ion and pH homeostasis [2,16]. If these four diverse CP-CSF functions are maintained in human elderly and Alzheimer’s patients, since there are no known alternative compensating mechanisms at the BCSFB, such data would strongly intimate that the fundamental CP transporters are functioning normally. Before reviewing the human data, a brief overview of these transport systems and comments on CSF production will put the human data in better perspective.

### AA transport into brain via CP

Hammarstrom, based on elegant autoradiographic studies of ^14^C-ascorbate distribution in mice, first proposed that AA was transported into CSF, and then brain, via the CP [6,17,18]. We confirmed and extended this hypothesis in rabbits [6,17,18]. AA does not enter brain directly through the capillaries of the BBB. In humans, there is also strong evidence for CNS penetration via CP-CSF rather than brain capillaries [18]. Subsequent work showed that the key AA transport system in CP is the sodium-dependent vitamin C transport system (SVCT2). SVCT2 is present on the basal (blood) side of the CP epithelium and neurons [19]. In vivo, in mammals including humans, the CP efficiently pumps AA from the blood flowing through the CP into CSF with a step-up in concentration of ~ 4 times [17-19]. In neurons AA is concentrated by another factor of ~50 by SVCT2 in the neuronal plasma membrane [19]. Thus, the concentration of AA in human plasma, CSF and brain neurons is ~ 50, 200, and 10,000 μM; and in whole brain the concentration approaches several mM [19]. The K_T_ (one-half saturation concentration) for SVCT2 is ~50 μM [18,19]. The correctness of this model is corroborated by the phenotype of SVCT2 knock-out (KO) mice and by the finding of multiple investigators that there is no SVCT2 protein in brain capillaries [19].

For details of the AA system in CP, there are many excellent reviews [19-21]. Still unknown is how AA exits the CP into CSF. But what is clear is how this elegant system maintains AA homeostasis in the central nervous system [6,18]. At low plasma concentrations, relatively more AA is pumped into CSF via the CP; at high concentrations, relatively less. In guinea pigs (which like humans cannot synthesize AA), the brain is the last organ to be depleted of AA in an experimentally-induced dietary AA deficiency state [6].

### Folate transport into CSF via CP

The folate transport system in CP works similarly to the AA system, actively pumping methyltetrahydrofolate, the principal folate in plasma, into CSF. Consequently, the steady-state concentration of methyltetrahydrofolate in CSF is ~2-4 times that of plasma [2,19,20]. The CP mechanism that transports folate from blood into CSF requires, in sequence, the folate receptor α (FRα) on the basal side of CP, the proton-coupled folate transporter (PCFT; SLC 46A1) and probably the reduced folate carrier (RFC; SLC 19 A1) at the apical side of CP [19,20]. There is evidently no FRα or PCFT in brain capillaries [19,20]. Naturally-occurring human KO’s of either FRα or PCFT lead to central folate deficiency states, very low CSF folate concentrations and severe neurological disease or death, establishing beyond doubt (in humans) the role of the CP in pumping folates from blood into CSF. From CSF, folates are transported into brain [19,20]. However, the exact mechanism(s) by which folates in CSF are transferred from human CSF into brain awaits elucidation. One group has postulated vesicular transport of folates out of mammalian CP through CSF and into brain [22]. However, in rabbits, over 70% of the CSF folate transferred from blood into CSF is present as free methyltetrahydrofolate not bound in vesicles [23]. We think that non-vesicular CSF folate transport occurs in humans since otherwise it would not be possible to measure CSF folate by current methods were it mainly bound in vesicles. However, the proportion of vesicular (if any) vs. non-vesicular transport of folate via human CSF remains to be experimentally determined.

### Transthyretin synthesis and secretion by CP

Transthyretin (TTR) is a protein of 508 amino acids [7]. Although the CSF protein level is < 1% that of plasma, approximately 10% of CSF protein is TTR [2,7]. Greater than 90% of the TTR in CSF is synthesized within and secreted into CSF by the CP [7]. Fasting reduces serum TTR (synthesized in liver) while the CSF concentration remains stable [7].

### Ion transport and acid–base balance in CSF

Another major function of CP epithelial cells is to maintain the Na, K and H ion concentrations in CSF [2,9,24]. Choroidal transport proteins, such as the CSF-facing Na-K pump and the Na-K-Cl cotransporter [25], actively maintain CSF [K^+^] close to 2.9 mM (or mEq/L) and [Na^+^] about 150 mM. Acid–base transporters in the BCSFB stabilize CSF pH near 7.3 [9] and [HCO_3_^-^] at about 24 mM. Perturbations in plasma electrolytes are not mirrored in CSF due to: *i)* the diffusion restriction imposed by the BCSFB tight junctions and, *ii)* the transporter activity by CP epithelium that tightly controls ion concentrations in secreted CSF [9,26,27].

### CSF production rates in the elderly and Alzheimer’s patients

Because reduced CSF production could alter solute concentrations in CSF, we reviewed the extant data on CSF formation in the elderly and Alzheimer’s patients. In the elderly, several studies have measured CSF production rates compared to younger humans. In seven normal elderly and seven young adults, May et al. employed the invasive Masserman technique which involves withdrawing CSF and then waiting for CSF baseline pressure to be restored [28]. By this indirect approach, May et al. reported decreased CSF production in the elderly [28]. However, there are several issues with this un-validated, fluid removal technique in humans (see below). Alternatively and more recently, a small, non-invasive phase-mapping study by MRI found no difference in CSF production in the young versus elderly [29]. In another investigation, Nilsson et al. quantified a >2-fold diurnal variation in human CSF production employing MRI analysis [30]. Therefore due to variability in methodology and findings, we conclude that the human CSF production rate as a function of age requires further examination.

In a study comparing CSF production in 7 patients with Alzheimer’s disease versus Parkinson’s patients, Silverberg et al. reported that CSF production was substantially decreased in Alzheimer’s patients [31]. However, they used a modification of the un-validated Masserman technique withdrawing 3 ml of ventricular CSF. Moreover, the Alzheimer’s patients were subject to general anesthesia; the “control” Parkinson’s patients, “conscious sedation.” Anesthesia has marked effects on CSF pressure and production rates [2]. Therefore the CSF dynamics data generated by the Masserman approach are not definitive. In the description below on CSF composition in the elderly and Alzheimer’s patients, we offer indirect evidence that the CSF production rate in dementia patients is normal or near normal. This may bear on interpreting CSF solute concentrations. Even if CSF formation rate remains stable, however, there might be a slower CSF turnover rate in aging or dementia if CSF volume is significantly increased [9]. A reduced CSF turnover rate [15,32,33] could affect CSF concentration of passively-distributed substances (such as albumin) between blood and CSF [34], but awaiting elucidation is how altered turnover rate would affect (if at all) the level of substances actively distributed by carrier mechanisms in CP.

### AA in CSF and brain of elderly and Alzheimer’s patients

In a superb study using a validated, non-invasive technique with magnetic resonance spectroscopy (MRS), Emir et al. showed that in the elderly (mean age 76; N = 22) the AA concentration in occipital cortex was slightly higher than in young adults (mean age 20; N = 22) [35]. This MRS finding is consistent with an ample supply of AA to neurons from the CP-CSF nexus.

Three age-and sex-matched, controlled studies of CSF and plasma AA levels in Alzheimer’s patients were described in an excellent review by Bowman [36]. The mean data from all three AA investigations are summarized in Table 1. What is remarkable (Table 1) is that although the CSF AA concentration was 13% lower in the Alzheimer’s patients, the mean ratio of CSF/plasma AA was 33% higher. This is expected of such ratios if the CP active and carrier-mediated transport is functioning normally; by Michaelis-Menten transport kinetics as the plasma concentration declines, relatively more AA is pumped into the ventricles to maintain CSF (and secondarily brain) homeostasis. As noted in Table 1, the mean plasma concentration in the Alzheimer’s patients was 31% lower than in age and sex-matched controls, presumably due to less AA in their dietary intake. In a separate uncontrolled study of 32 Alzheimer’s patients, Bowman et al. found a mean CSF/plasma ratio of 4.0 ± 0.3 (SEM)– confirming the findings summarized in Table 1[37]. Collectively, these data provide no support for abnormal AA transport function in CP of elderly or Alzheimer’s patients. In fact, they strongly support the normal ability of CP in Alzheimer’s patients to actively concentrate AA in CSF.

**Table 1:** Mean plasma and CSF AA concentrations, and ratios of CSF/plasma AA concentrations in Alzheimer’s patients and controls*

**Assessment**	**Control**	**Alzheimer’s**
CSF AA (μM)	183	160
(N)	(43)	(47)
Plasma AA (μM)	68	47
(N)	(43)	(47)
Ratio of AA, CSF/plasma	3.0	4.0
(N)	(43)	(47)

*The data are weighted means of three age-and sex-matched, controlled studies (See Table 3 of Bowman [36] for details.) Number of subjects is in parentheses.

### CSF folate concentration in elderly and Alzheimer’s patients

In 36 elderly men and women (mean age 73), Serot et al. found no difference in the mean concentration of CSF folate versus that in 60 younger adults (mean age 40) [38]. However, in 36 Alzheimer’s patients, Serot reported a 17% mean decrease in CSF folate and an 8% mean decrease in plasma folate compared with the 36 elderly controls [38]. Although the CSF concentration of folate in Alzheimer’s patients was slightly lower than in aged controls, it was still within the normal range and not low enough to cause symptoms. Moreover, the CSF folate concentration in Alzheimer’s patients was still much higher than the plasma concentration. This reflects adequate CP functioning, i.e., maintenance of CSF folate against an uphill concentration gradient across the BCSFB. Others [39] have reported normal CSF folate concentrations in Alzheimer’s patients and showed, as expected, a negative correlation between the plasma folate concentration and the ratio of CSF/plasma folate (P 0.001). This confirms the normal, saturable operation of the folate transport system in CP in Alzheimer’s disease [19,20,23].

### CSF transthyretin in the elderly and Alzheimer’s patients

In lumbar CSF of patients with a normal level of CSF protein and less than three cells/ml of CSF, Serot et al. measured TTR [7]. As per Table 2, Alzheimer’s patients had a mean 13% decrease in CSF [TTR] vs. elderly controls, but a CSF TTR level comparable to middle-aged controls and higher than in young subjects. It is risky to interpret these data too finely, but clearly Alzheimer’s patients have a CSF TTR concentration within the normal range. This strongly implies adequate CP secretory function.

**Table 2:** Transthyretin (TTR) concentration in lumbar CSF*

**Group (mean age)**	**Mean TTR (mg/L) ± SD (N)**	**% control**
Age-matched controls (76)	20.0 ± 2.5 (41)	
Alzheimer’s patients (74)	17.5 ± 2.0 (40)	87
Middle-aged (41)	17.4 ± 2.4 (51)	87
Youth (10)	15.5 ± 1.8 (17)	78

*Data are compiled from Serot et al. [7].

Moreover, Serot et al. also measured CSF albumin in these subjects [7]. The mean concentrations of CSF albumin in the middle-aged controls (N = 51), elderly (N = 41) and Alzheimer’s patients (N = 40) were 0.21, 0.22, and 0.22 g/L, respectively. These similar CSF albumin concentration data fit the notion that there is no appreciable loss of integrity of the blood-CSF barrier with aging and Alzheimer’s disease. This conclusion seems likely because increased CP permeability in dementia would presumably have caused an increased concentration of CSF albumin. Moreover, deductively, there is probably no substantial decrement in CSF production since a lower fluid turnover rate would also tend to increase the CSF albumin concentration. Others have reported CSF albumin concentration stability in Alzheimer’s disease [39].

### Stability of CSF electrolytes and pH in Alzheimer’s disease

In Alzheimer’s subjects, there is compelling evidence for unaltered concentrations of CSF [Na^+^] and [K^+^] compared to young subjects and age-matched controls [40] (Table 3). This is in line with the upregulated Na-K-Cl cotransporter protein in human CP in Alzheimer’s disease [9,13]; this apically-located cotransporter, along with the Na-K exchange pump, has a putative role in CSF inorganic ion homeostasis by adjusting ion fluxes across the CSF-facing membrane of choroid epithelium [25]. Similarly, Alzheimer’s patients had normal CSF acid–base balance (pH = 7.33) and pCO_2_ levels [41,42] (Table 3). Such CSF acid–base stability in the elderly and Alzheimer’s subjects points to compensatory functioning of HCO_3_^-^ and H^+^ transporters at the BCSFB. Accordingly, although the information is somewhat limited, the available evidence (CSF ion composition data from 30 Alzheimer’s patients and 50 control or normal subjects) strongly suggests that regulatory Na^+^, K^+^ and H^+^/HCO_3_^-^ transport at the BCSFB is maintained in Alzheimer’s disease [40-42].

**Table 3:** **[Na**^**+**^**], [K**^**+**^**], [HCO**_**3**_^**−**^**], pH and pCO**_**2 **_in CSF of normals, controls and Alzheimer’s patients*

Ion**	**Normal values**^**≠**^	**Controls**	**Alzheimer’s**
[Na^+^]	------	146 ± 2 (15)	147 ± 2 (15)
[K^+^]	------	2.8 ± 0.1 (15)	2.8 ± 0.1 (15)
[HCO_3_^-^]	22.9 ± 2.3 (35)	------	23.4 ± 1.3 (15)
pH	7.31 ± 0.03 (35)	------	7.33 ± 0.03 (15)
pCO_2_ (mm Hg)	47.9 ± 5.7 (35)	------	46.9 ± 3.2 (15)

^≠^Normals = normal adults; controls = age-and sex-matched elderly.

*[Na] and [K] data are compiled from Vitvitsky et al. [40]. Acid–base data are obtained from Posner et al. [42] (normal values) and Gottfries et al. [41] (controls and Alzheimer’s patients).

** Ion concentrations are in mEq/L.

≠ All values are means ± SD; N = number of subjects in parentheses.

## Discussion and conclusion

The collective observations for CP-CSF function in four different complex energy-requiring transport and homeostatic systems (AA and folate transport into CSF via CP, TTR synthesis/secretion into CSF by CP, and impressive ion and pH stability in CSF) strongly support the notion that the CP in elderly and Alzheimer’s patients generally functions well. This is in the face of clear histological, pathological and anatomical modifications in CP tissue of some elderly and Alzheimer’s disease patients [1,12,13,31]. Thus, the hypothesis of compromised CP homeostatic transport failure in some elderly and Alzheimer’s patients, postulated earlier by us and others [11-13], is not supported by the CSF compositional evidence reported in this review. Clearly there is more work to be done such as resolving CSF production rates using validated techniques taking diurnal variation, anesthesia and other factors into account. However, the human CSF data reported herein by us and others substantiate the maintenance of CP transport and homeostatic functions in the elderly and Alzheimer’s disease.

## Abbreviations

AA: Ascorbic acid; Alz’s: Alzheimer’s disease; BBB: Blood–brain barrier; BCSFB: Blood-CSF barrier; CP: Choroid plexus; CSF: Cerebrospinal fluid; KO: Knock out; Na-K-Cl cotransporter: Sodium-potassium-chloride cotransporter in CP; PCFT: Proton-coupled folate transporter; RFC: Reduced folate carrier; SVCT2: Sodium-dependent vitamin C transporter 2; TTR: Transthyretin.

## Competing interests

The authors declare they have no competing interests.

## Authors’ contributions

Both RS and CEJ analyzed the literature and wrote the manuscript. Both authors have read and approved the final manuscript.
